# Supplementary material for: Chromothripsis during telomere crisis is independent of NHEJ, and consistent with a replicative origin
Source: Genome Res. 2019 May;29(5):737–49. doi: 10.1101/gr.240705.118 (PMC6499312; doi:10.1101/gr.240705.118)
Supplement: Supplemental Material [file supp_gr.240705.118_Supplemental_file_1.zip › contigs/annotated_contigs/DB57/contig.5.DB57_length_1481_mean_cov_15.1100607698.docx]

**DB57_length_1481_mean_cov_15.1100607698**

```
GCCCGAACACCTGAACCCAACACCGCGCAAAAGAACGAATCTAACTCA|AAAAAAAAAAAAAAAAAAAATAACCAACTATCATATAAAT
                                                 >chr7:146028395-146028641 + E=9e-133 p=
TATTCGGCCCTTTATTGAGTATATTCCATTTATCCAGCAGTGTGATTTCTGGCTGTCTCAGTTATTCAGTATATAGGCTCAGATCAGAG
5e-02
TAAGCAGAAGAGGACTTTGAACATCAGAGAGCAGTCAGAACTGGAGAATCTACTTGCAGATGAGGGATTTATTACAGGTCTAGTGACTC

ATTGCTGTTTGGTATCTCATCCTCCA|GG|AGTGCAGTGGCGTGATCATGGCTCACTGCAACCTCTGCCTCCTGGGCTCGAGTGATCCT
                           >chr7:147215071-147215606 + E=2e-309
CCCACCTCACCGTGTCTGTTTTGTTTAAGAGTATATAGTTATCAATCCTTAAAGCCGAATTTCATTCTTGGTAGTTACTGTTAAAATTA

AGCATGAACAATTGAAAGATTTTACAAAGTATATCTATCATATACCTGCGGTGTATCATCCTTGGAGAAGAAATGAACATACACTGAAC

ATAGAAACAACTAAACTTTCTAGATAAATGGAACTAAGAGACTAACTTCTGTTTCAAATGATGACGAATTTCAGAAAGAGAAAAAATTA

AGTAGAAAAAAAAGGAGAATGTGAGAGACTGGGGAGAGAAAAAATAAATGAGAGAAGGTGGTAGACCACTAGGATGAAGAAGCAGAAGA

TGAAGGAGACAGGCAAGATGCTGACCTCTTAGCTCAGTCAAGGTCTGGACTTTGGGGGCCTGTCCTAGCATCATCTCTTGCAGTCTAGC

ACATGTACATACAGATGCTATGAGAACAT|TTTTAAAAGATTT|TTTCTTAGTGCCTTATCAAAGAGAAGCCTTCTTGGGCACCATTAG
                                           >chr7:141195250-141195387 + E=3e-70
TGGGTGGTAAATTGGTGGGGGTGCTTTAGAGGACAACTTGACAATATCAAGATTTGAAAGGCACTTGCAGAAATTCCCTTTCTGGAAA|

TTTT|ATAAGGTCTCTTTTCAGGGTGGTTGA|AATTTTTAAAAG|ATTTCCTTGTCTGCTATCCTCATAACTGGGACTCCCTTTATTTC
>chr7:147215804-147215834 - E=6e-06       >chr7:147207111-147207577 - E=5e-268
TGAAGTCCTTTGTCAGTGTATTCTGCAGGGATCTCCTTGACTATGTCCTCTGTCCTGGTGAGGGTGAAGAGACTGAAACTCTTGATCAT

GAGGTAATGGAAAGTCTGCATGGCTGGTGGCTTACATGGCAAGGATAAGGTGGAATGGCCAGCCAATTTGCTCTGTAAAAGGCCACTAA

ATACTGGGTTTTAGCCCTCAGAGTCTGGGGAACTTGAAGTCAAAGAAGAGAAAGAATTAGAATACGCAGAACCCCAGACAGGTTTCATC

TTCTGATCAGATAGAAAGGCTAAGAGAGAAAACAGATTCCCTAGTCACACTGGCACGAATCCTCTGCTCTGGAATCTTCTCATAAGCCC

AGTGAAATTCAGATTCCATGACTCTATTATAGACCTTATGAATCAGAAGGGATGGTGCCCAGGAGT
```
